# Supplementary material for: Phytochemical Profiling, In Vitro Biological Activities, and In-Silico Studies of Ficus vasta Forssk.: An Unexplored Plant
Source: Antibiotics (Basel). 2022 Aug 26;11(9):1155. doi: 10.3390/antibiotics11091155 (PMC9495161; doi:10.3390/antibiotics11091155)
Supplement: Supplementary file 1 [file antibiotics-11-01155-s001.zip › antibiotics-1826368-supplementary.pdf]

antibiotics

Supplementary file

Phytochemical profiling, *In vitro* Biological activities, and *In-Silico* Studies of *Ficus vasta Forssk.*: An unexplored plant

Hanan Y. Aati [1,*,†], Mariyam Anwar[2], Jawaher Al-Qahtani [1], Areej Al-Taweel [1], Kashif-ur-Rehman Khan[2,*,†], Sultan Aati[3], Faisal Usman[4], Bilal Ahmad Ghalloo[2], Hafiz Muhammad Asif[5], Jafir Hussain Shirazi[6], Aliza Abbasi[2]

1 Department of Pharmacognosy, College of Pharmacy, King Saud University, Riyadh 11495, Saudi Arabia
2 Department of Pharmaceutical Chemistry, Faculty of Pharmacy, The Islamia University of Bahawalpur, Bahawalpur 63100, Pakistan
3 UWA, University of Western Australia, Nedland, WA 6009, Australia
4 Department of Pharmaceutics, Faculty of Pharmacy, Bahauddin Zakariya University, Multan 60000, Pakistan
5 Faculty of Medicine and Allied Health Sciences, University College of Conventional Medicine, The Islamia University of Bahawalpur, Bahawalpur 63100, Pakistan
6 Department of Pharmaceutics, Faculty of Pharmacy, The Islamia University of Bahawalpur, Bahawalpur 63100, Pakistan
* Correspondence: hati@ksu.edu.pk (H.Y.A.); kashifur.rahman@iub.edu.pk (K.-u.-R.K.)
† These authors contributed equally to this work.

Citation: Aati, H.Y.; Anwar, M.; Al-Qahtani, J.; Al-Taweel, A.; Khan, K.-u.-R.; Aati, S.; Usman, F.; Ghalloo, B.A.; Asif, H.M.; Shirazi, J.H.; et al. Phytochemical profiling, In vitro Biological activities, and In-Silico Studies of *Ficus vasta Forssk.*: An unexplored plant. *Antibiotics* **2022**, *11*, 1155. https://doi.org/10.3390/antibiotics11091155

Academic Editor: Muhammad Jawad Nasim

Received: 5 July 2022
Accepted: 23 August 2022
Published: 26 August 2022

Publisher's Note: MDPI stays neutral with regard to jurisdictional claims in published maps and institutional affiliations.

Copyright: © 2022 by the authors. Submitted for possible open access publication under the terms and conditions of the Creative Commons Attribution (CC BY) license (http://creativecommons.org/licenses/by/4.0/).

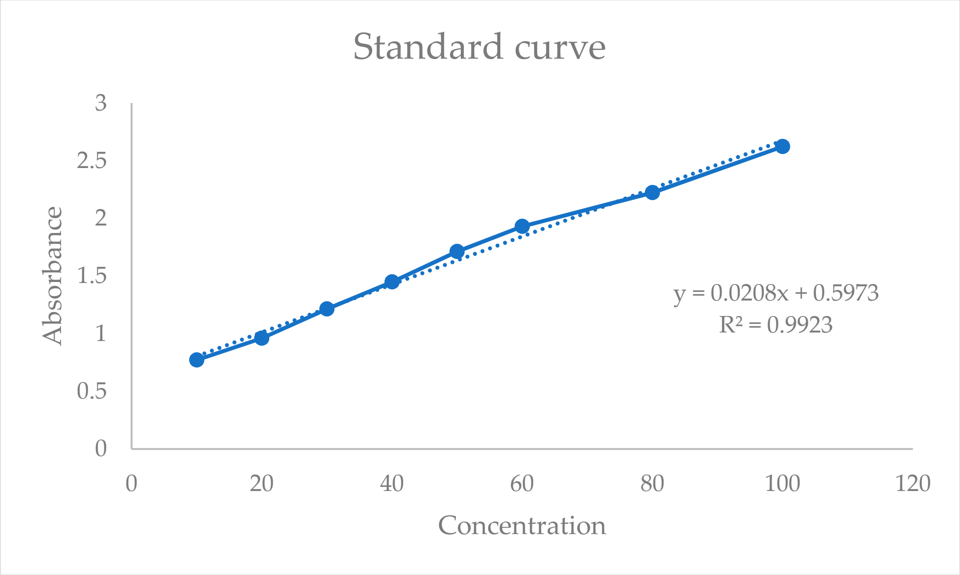

Figure S1. Standard curve, regression equation and R^2 for quantification of phenolics

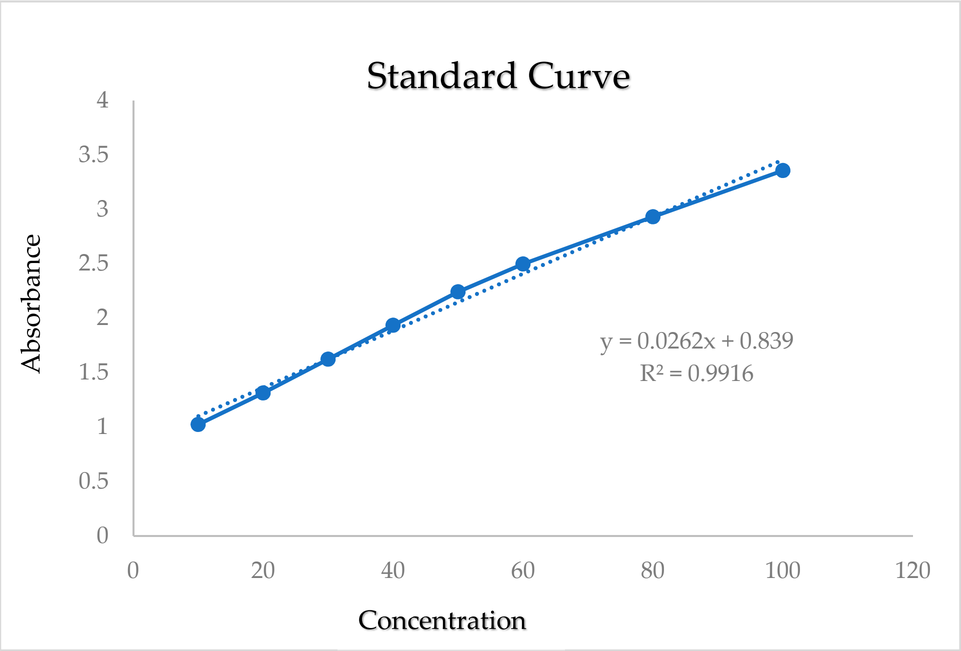

Figure S2. Standard curve, regression equation and R^2 for quantification of flavonoids

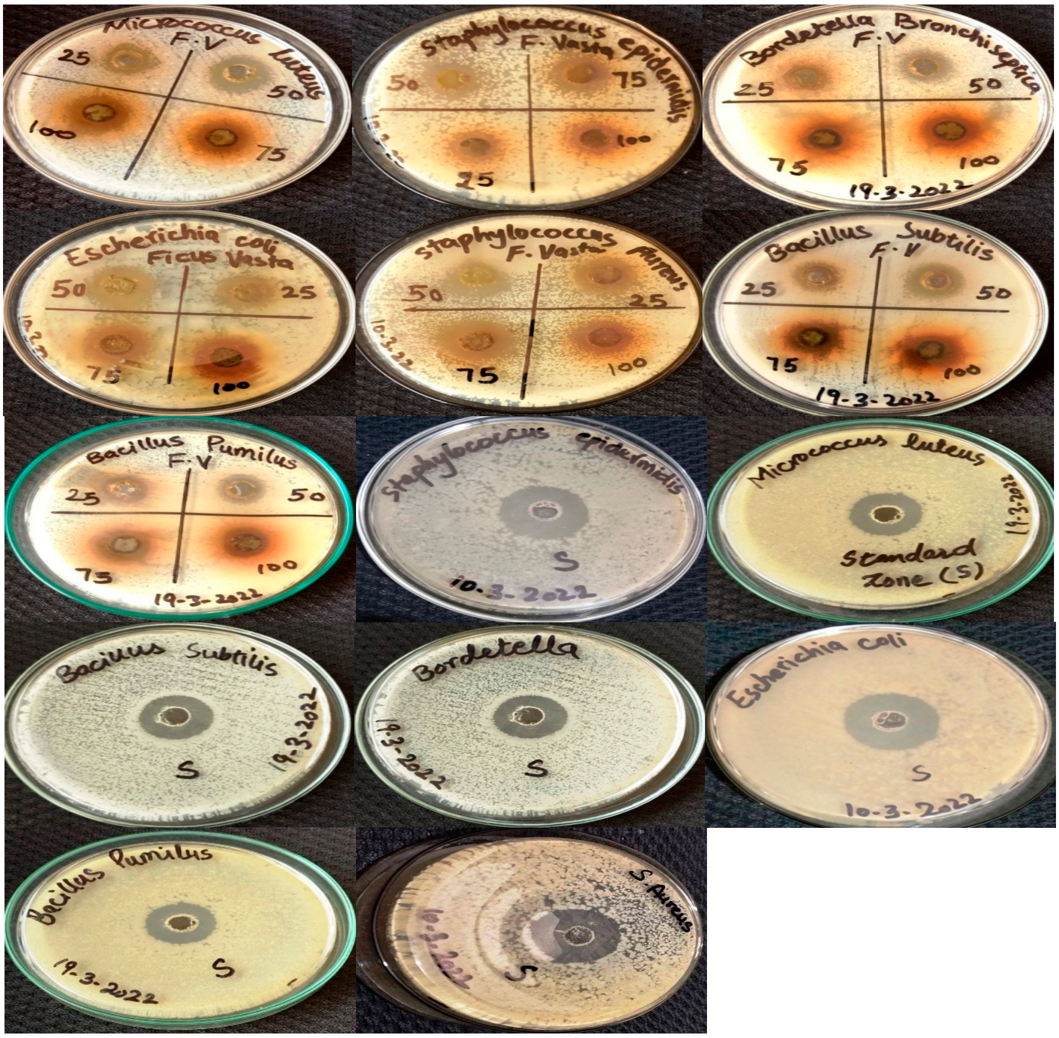

Figure S3. Antibacterial results of *F. vasta* ethanolic extract.

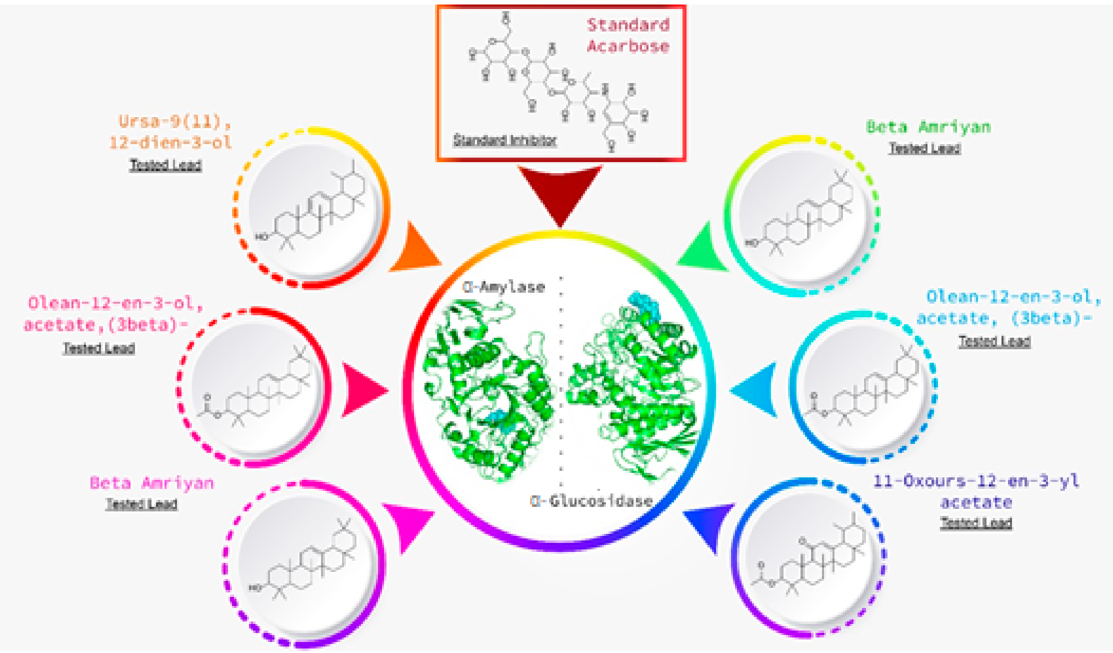

Figure S4. Graphical representation of best three docked compounds and Acarbose against α-glucosidase and α-amylase.

Table S1. Pharmacokinetic properties of best-docked compounds.

Sr no.	Best docked compounds	Gastrointestinal absorption	Blood-brain barrier	P-glycoprotein substrate	CYP inhibitors					log Kp skin permeation (cm/s)
					CYP 1A2	CYP 2C19	CYP 2C9	CYP 2D6	CYP 3A4	
1	Ursa-9(11),12-dien-3-ol	Low	×	×	×	×	×	×	×	-2.69
2	Olean-12-en-3-ol, acetate, (3beta)-	Low	×	×	×	×	×	×	×	-2.25
3	Beta-Amyrin	Low	×	×	×	×	×	×	×	-2.41
4	11-Oxours-12-en-3-yl acetate	Low	×	×	×	×	×	×	×	-3.2
5	Campesterol	Low	×	×	×	×	×	×	×	-2.5
6	Beta-Sitosterol	Low	×	×	×	×	×	×	×	-2.2
7	Stigmasterol	Low	×	×	×	×	✓	×	×	-2.74

×; NO, and ✓; yes.

Table S2. Toxicity profiles of best-docked compounds.

Sr no.	Best docked compounds	LD50 mg/kg	Predicted class	Hepatotoxic	Carcinogenic	Immunotoxic	Mutagenic	Cytotoxic
1	Ursa-9(11),12-dien-3-ol	288	3	×	×	✓	×	×
2	Olean-12-en-3-ol, acetate, (3beta)-	3460	5	×	✓	✓	×	×
3	Beta-Amyrin	70000	6	×	×	✓	×	×
4	11-Oxours-12-en-3-yl acetate	3300	5	×	✓	✓	×	×
5	Campesterol	890	4	×	×	✓	×	×
6	Beta-Sitosterol	890	4	×	×	✓	×	×
7	Stigmasterol	890	4	×	×	✓	×	×

✓: Active, ×: Inactive, Class I: $LD_{50} \leq 5$, class ii: $5 < LD_{50} \leq 50$, class iii: $50 < LD_{50} \leq 300$, class iv: $300 < LD_{50} \leq 2000$, class v: $2000 < LD_{50} \leq 5000$, and class vi: $LD_{50} > 5000$.

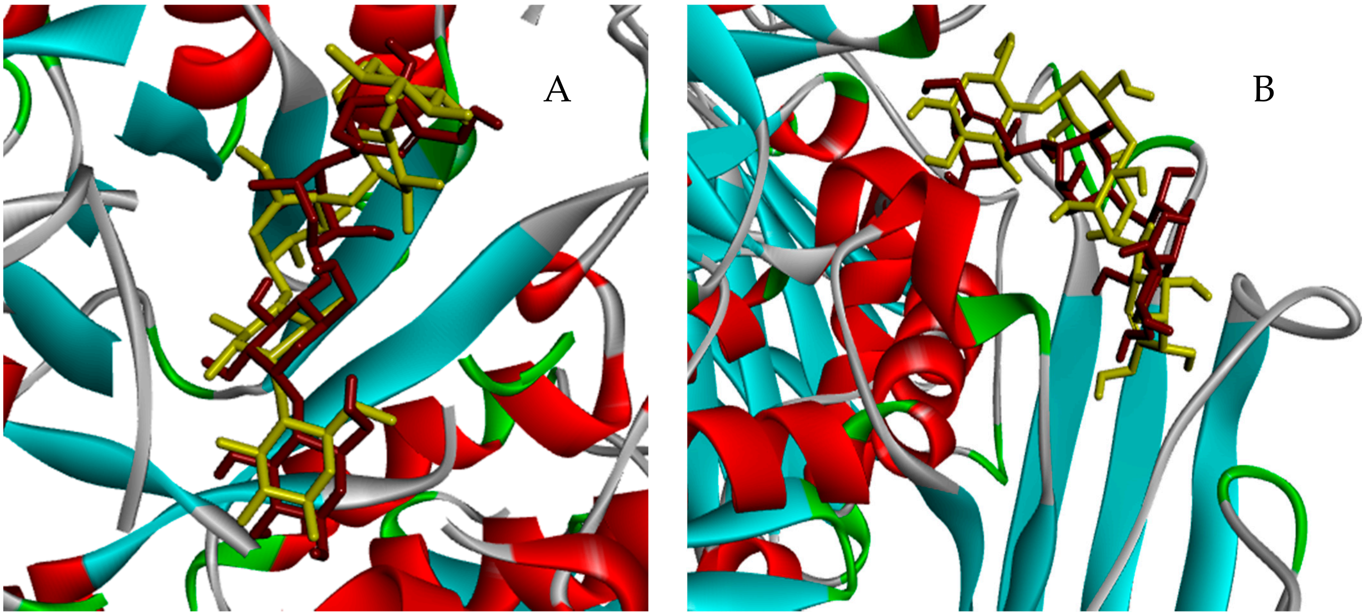

Figure S5. Superimposition of re-docked (Red) onto co-crystallized Acarbose (Yellow) in the active site. "A" α-amylase (RMSD value=1.525 Å) and "B" α-glucosidase (RMSD value=1.234 Å).
